# Duplication of the transverse colon in adults: a case report and literature review

**DOI:** 10.3389/fonc.2023.1230860

**Published:** 2023-09-04

**Authors:** Xiaochun Zhang, Guangci Di, Wei Cheng, Cuizhong Wang, Guanwen Gong, Zhiwei Jiang

**Affiliations:** ^1^ Department of General Surgery, Affiliated Hospital of Nanjing University of Chinese Medicine, Nanjing, Jiangsu, China; ^2^ Department of Gynecology, Affiliated Hospital of Nanjing University of Chinese Medicine, Nanjing, Jiangsu, China

**Keywords:** transverse colon, duplication of deformity, abdominal pain, adult, space-occupying lesion, surgical resection

## Abstract

**Background:**

Duplication of the transverse colon is a rare gastrointestinal malformation. Its pathogenesis is still unclear, and it is extremely rare in adults. Patients often present with symptoms of tumor compression such as abdominal mass, abdominal pain, and constipation as the first manifestation.

**Methods and result:**

A patient with a duplication of the transverse colon was admitted to the Department of General Surgery of our hospital. Laparoscopic exploration found a mass at the rear of the transverse colon near the splenic flexure, and the root was connected to the middle portion of the transverse colon.

**Conclusion:**

Surgery is a radical treatment and reduces the possibility of perforation, bleeding, obstruction, and cancer.

## Introduction

**Figure 1:**
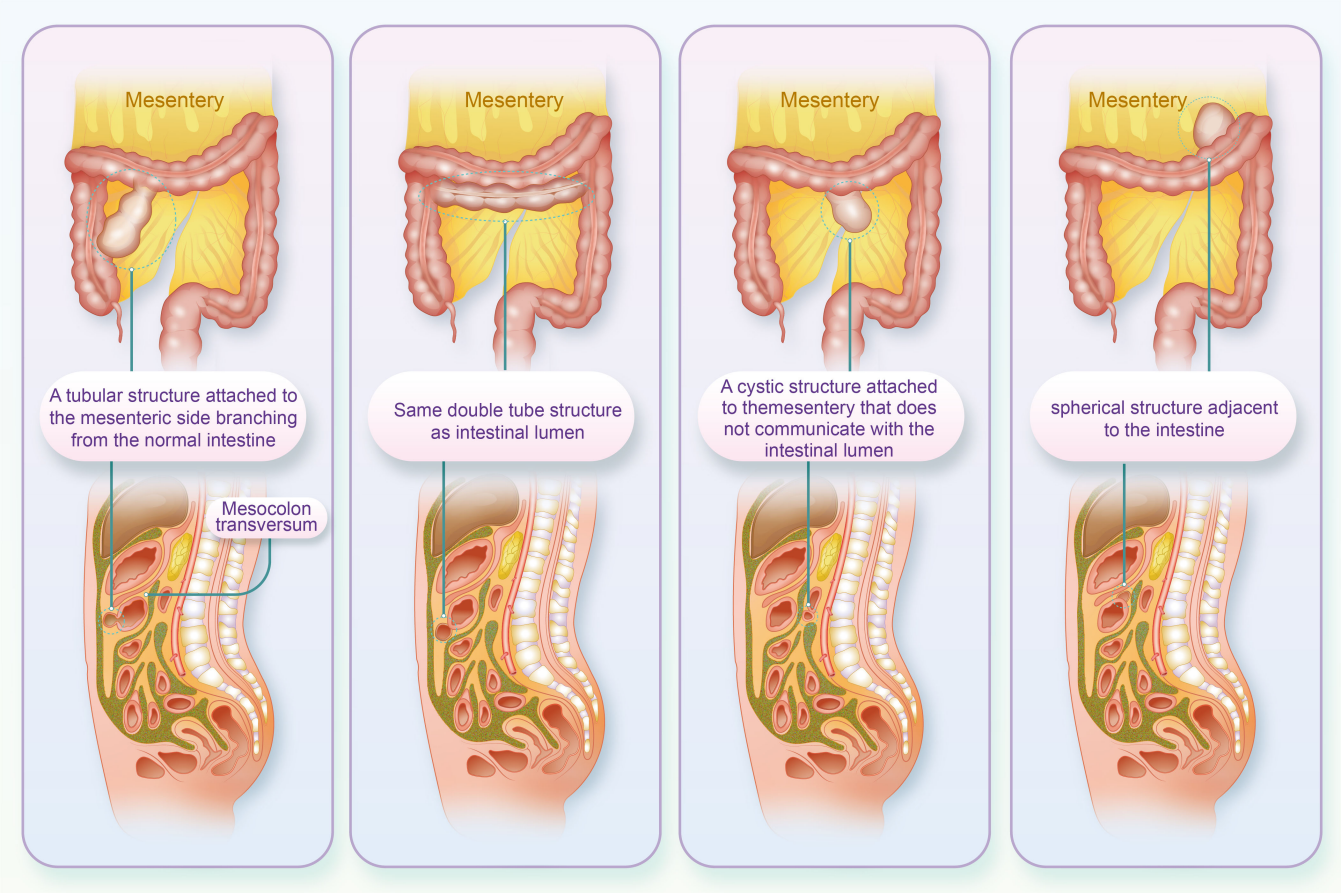
The four structures of the intestinal repeat (Above is the coronal position, below is the sagittal position).

Intestinal duplication malformation is a rare congenital gastrointestinal malformation clinically known as ileal and ileocecal valve malformations. The condition is more common in children under the age of two, with the most common type being ileal and ileocecal valve malformations (approximately 60%), followed by the jejunum (8%), colon (6%) and rectum (5%). Intestinal repeats can generally be divided into four structures (1): tubular structure attached to the mesenteric side separated from the normal intestine (2); double-tube structure communicating with the intestinal lumen (3); cystic structure attached to the mesentery and not communicating with the intestinal lumen; and (4) spherical structure adjacent to the intestine (**Figure 1**) (1). The cystic structure is the most common, accounting for 90%-95%, while the tubular structure accounts for 5%-10%. The symptoms of this condition are nonspecific and can include abdominal mass, pain, constipation, intussusception, bleeding, and other manifestations. As a result, it can easily be misdiagnosed as an abdominal tumor (2). There have been few reports of repetitive malformations in the transverse colon in China. On January 28, 2023, a patient with a duplication of the transverse colon was admitted to the Department of General Surgery of our hospital in order to improve the understanding of the disease among gastrointestinal surgeons and include it in the differential diagnosis of abdominal pain.

## Case presentation

**Figure 2:**
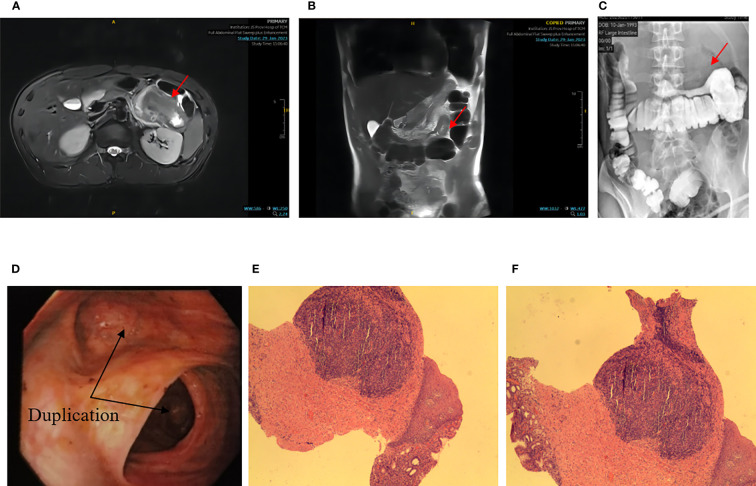
**(A)** NMR transverse axial view: the red arrow indicates a soft tissue mass located in the left mid-abdomen, closely related to the bowel, approximately 6.4*4.7cm, with multiple internal gas signals; **(B)** Coronal view of MRI; **(C)** Barium enema X – ray: the red arrow points to a right-to-left image of a smaller intestinal tube about 190mm long; **(D)** Endoscopy: the black arrow points to an open-like structure, and the distal segment’s stenosis was obvious; **(E, F)** Pathology: chronic inflammation of mucosal tissue covered with squamous epithelium and gastric columnar epithelium). Coloration HE, magnification ×100.

On January 2, 2023, an unmarried 30-year-old male patient presented with intercropping epigastric distension and pain that had persisted for over a month and worsened over the past week. One month prior, the patient experienced epigastric tenderness without being predisposed to referred pain, nausea, vomiting, acid belching, or hiccups. 2023 January 26 to another hospital to check the abdominal computed tomography (CT) suggests: “The left middle abdominal soft tissue mass is occupied, considering the malignant tumor lesion, there is pipeline communication between the lesion and the transverse colon, and the gas accumulation is seen in it, the other end is the blind end, and the periphery of the lesion exudes, and multiple enlarged lymph nodes can be seen”. After rehydration, pain relief were administered. However, there was no significant improvement, and the patient was transferred to our hospital to enroll in the “abdominal pain” department. Upon admission, the patient complained of obvious swelling pain and occasional colic with cold sweats, but there was no cough or eating obstruction. The patient reported poor sleep, normal urine adjustment, and 2-3 bowel movements per day, with no black or bloody stool. There was no previous family history of intestinal congenital diseases. There was no previous history of abdominal surgery. Physical examination revealed a flat abdomen with no gastrointestinal type, peristaltic waves, mid-upper abdominal tenderness, rebound tenderness, and no palpable mass. After a complete 3.0 T full abdominal magnetic resonance (MRI) plain scan and enhancement, the patient was diagnosed with a soft tissue mass in the left middle abdominal tube with a size of approximately 6.4*4.7 cm. The mass showed a long T1 and long T2 signal, and multiple gas signals were seen inside the lesion. The sac-like long T1 long T2 signal shadow was seen behind the left peritoneum, the size was approximately 2.1*1.3 cm, and the enhanced scan did not show strengthening. The results suggest the presence of left mid-quadrant lesions, which could be inflammatory lesions or lymphoma that need to be drained. Moreover, there was a left retroperitoneal cystic mass, which could be a benign lesion, but lymphangioma was possible, and regular review was recommended. In the case of barium enema colitis, the upper right edge of the transverse colon is seen to the right to left, with a walking curved thin bowel tube-like shadow. The base is approximately 42 mm wider, and in the supine position, it is inflated, the prone position is full, the liquid level is visible in the distal section of the vertical position, the total length is approximately 190 mm, the blind end is seen, and a titanium clip can be seen in the proximal section. The results suggest an abnormal open bowel lesion in the transverse colon. A painless colonoscopy revealed a dilated lumen and an open-like structure in the reverse scope. Pathological diagnosis showed chronic inflammation of mucosal tissue covered with squamous epithelium and gastric columnar epithelium (**Figures 2A–F**).

**Figure 3:**
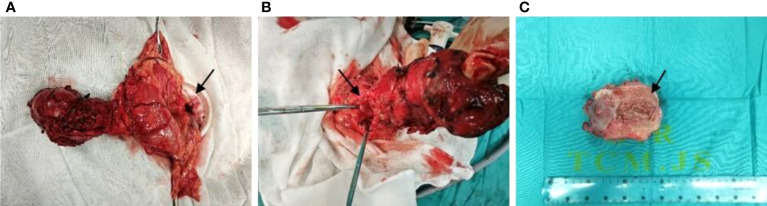
Specimens seen and resected during surgery [**(A, B)** proximal splenic flexural mass at the back of the transverse colon, approximately 8.0 cm by 9.0 cm, inflammatory edema of the surrounding tissues, the root of the mass is connected to the middle section of the transverse colon, and the root of the mass is exposed with nourishing blood vessels emanating from the ligament of Treitz; **(C)** The specimen showed intestinal mucosal tissue in the mass. The black arrow indicates a duplicate deformity that separates from the normal transverse colon and communicates with the intestinal lumen)].

To confirm the diagnosis, laparoscopic surgery was performed under general anesthesia at 1:40 on February 7, 2023. Intraoperative exploration revealed a palpable mass near the splenic flexure at the back of the transverse colon, measuring approximately 8.0 cm by 9.0 cm, with an intact capsule, inflammatory edema of surrounding tissues, and adhesions. The mass did not invade adjacent organs, and the root was connected to the middle section of the transverse colon, confirming the preoperative diagnosis of repetitive deformity of the transverse colon. The root of the mass was exposed, revealing nourishing blood vessels emanating from the ligament of Treitz, and a dissected specimen showed the presence of intestinal mucosal tissue in the mass (**Figures 3A–C**). we removed the duplicate colon and extended it 2cm into the original transverse colon, and performed a colon-colon anastomosis with a stapler. After surgery, the patient underwent an accelerated rehabilitation program and recovered well, leading to their discharge from the hospital on February 13.

**Figure 4:**
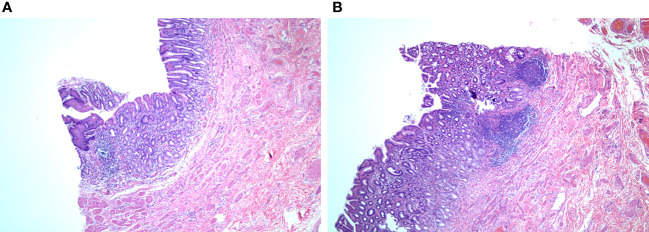
Pathology report **(A)** and **(B)** can be seen in the intrinsic muscular wall, covered with squamous epithelium, gastric mucosa, and large intestine mucosa). coloration HE, magnification ×200.

Postoperative pathology (**Figures 4A, B**) (transverse colon) revealed a microscopic organ-like structure visible to the intrinsic muscle wall, which was covered with squamous epithelium, gastric mucosa, and large intestine mucosa. The propria muscle wall and extramuscular fibrous tissue showed hyperplasia with focal inflammatory granulation tissue hyperplasia, foreign body giant cell reaction, and abscess formation. Combined with clinical findings, this is consistent with repetitive digestive tract malformations with chronic active inflammatory changes. Immunohistochemical results indicated that hyperplastic fibrous tissue expressed SMA (+), Desmin (focal+), S-100 (-), cald esmon (-), B-catenin (pulp+), and CD34 (vascular +).

## Discussion

Gastrointestinal repetitive malformations are rare congenital malformations and usually present as cystic or tubular structures on one side of the mesangium in neonates (3, 4). The incidence of the disease is 1/10000~1/4500, and it can occur in the entire digestive tract from the oropharynx to the anus (5). Colonic duplication is a type of repetitive gastrointestinal malformation reported in fewer than 50 cases in the literature since 1950, with 80% of cases occurring before age 2 (6). Adult onset of the disease is rare (7). The disease was first reported in 1876 and is known as “alimentary tract duplications” (8). The disease has three major features: (1) it is similar to normal intestinal structure; (2) the lining mucosa is similar to the intestine; and (3) it is attached to the normal intestinal tract and shares blood supply (9). The disease does not have specific symptoms, but it may cause an abdominal mass, chronic pain, and constipation. Volvulus, intussusception, bleeding, or perforation are rare and generally occur in the sigmoid colon, similar to diverticulitis. Colonic repetitive malformations can occur anywhere in the colon, mostly with normal digestive tract structure. Most malformations fuse with the attached main bowel to form a common muscular wall, sharing a common serosus, intestinal membrane, and blood supply, but with independent, separate, or communicating mucosal cavities. For repetitive colonic malformations, McPherson AG (10) proposed another classification, namely, type I (simple cyst), type II (diverticulus), and type III (tubular colon malformation) (**Table 1**), which in turn can be Y-type and double-cast (11). In this case, the root of the mass is connected to the middle section of the transverse colon, the proximal blind closure. A large amount of mucosal secretion accumulates in the intestinal lumen, which belongs to type III. Y type.

**Table 1:** Classification provided by McPherson AG.

Type	position	structure	Blood supply	clinical feature
I	In the mesentery of the colon, either next to or away from the intestinal wall	Composed of intestinal epithelium	The blood supply is inseparable from that of the adjacent intestinal canal.	Palpable masses or intestinal obstruction
II	The margin of the mesentery or the reverse mesentery	The lumen of the diverticulum is lined with intestinal mucosa, but not necessarily of the colonic type.	Blood supply is intertwined with that of the adjacent colon.	A mass in the abdomen
III	A long repetitive malformation with the duplicated bowel partially parallel to the normal bowel	Normal mucosal lining	Consistent with the blood supply to the adjacent intestine	If both colons can empty freely, there can be no symptoms.

Based on the literature at home and abroad to clear diagnosis of adult transverse repeat deformity in 16 cases and 1 case analysis, this paper in order to improve the understanding of the disease (**Table 2**) (12–26). Two main theories explain the pathogenesis of repetitive malformations in the transverse colon. The first theory, abnormal lumen recanalization, suggests that during embryonic development, the internal embryonic vacuole enlarges and fuses to form the intestinal lumen. However, a range of factors can cause the vacuole to remain separated, resulting in one or more primitive intestinal repeats. The second theory, the diverticular theory, proposes that embryonic diverticula cause repetitive malformations. However, this theory cannot fully explain complete pancolonic repetitive malformations with a round or longitudinal muscular layer (27, 28). In addition, environmental factors such as trauma or hypoxia can contribute to the condition (29). Over time, the compressive effects due to secretion and accumulation of intestinal mucus may be the underlying pathogenesis.

**Table 2:** Demographic and clinical profiles of transverse colon duplication have been reported previously.

Author	Year	Sex	Age	Location	type	symptom	Preoperative diagnosis	accessory examination	therapy method	Follow up (month)
JIANG Jiaquan et al. (12)	2022	M	34	Middle transverse colon	III	No	Transverse colon cancer	CT: uneven thickening of intestinal tube wall at transverse colon-descending colon transition	Surgery: transverse colon resection	6
KE Feng et al. (13)	2014	M	45	Middle transverse colon	III	Abdominal mass, abdominal pain	Giant teratoma	US: Form right kidney disorders; CT: Abdominal teratoma possible; Barium enema: A fissure in the transverse colon associated with the mass	Surgery: repeat segment colon resection, transverse colon sigmoid anastomosis	N/A
Siamionava Y et al. (14)	2019	F	18	The origin transverse mesocolon	III	Chronic constipation, flatulence, repeated episodes of abdominal pain	Mega caecum	US: pronounced flatulence; Barium enema: right part colon elongation revealed, occupying two-thirds of the abdominal cavity.	Surgery: transverse colon resection with duplication and descending colon with ascendosigmoid anastomosis creation	N/A
Banchini F et al. (15)	2013	M	21	The origin transverse mesocolon	III	Chronic constipation, abdominal pain	Intestinal duplication	US: not diagnostic; CT: an oval mass with wall enhancement and similar stool material inside	Surgery: the duplicate intestine and original transverse colon were removed by 5cm and a colo-colon anastomosis was performed.	N/A
Bellanova G et al. (16)	2015	F	39	Anti-mesenteric side of transverse colon	I	Upper abdominal pain	Inflammed Meckel’s diverticulum	Plain abdomen X-ray: no significant findings; US: 55 mm mesogastric cystic lesion; CT: a 55 × 45 mm, smoothly rounded, cystic lesion located between the duodenum and the gallbladder, close to the transverse colon	Surgery: the cystic lesion was cut off and the mass was completely removed	30
Otomi M et al. (17)	2013	M	26	Middle transverse colon	III	Abdominal pain, fever	Diverticulitis	CT: the intestinal wall is swollen and hypertrophic, and a large number of hypodense shadows can be seen inside; Barium enema: in the middle of the transverse colon, contrast media leaks out of the bowel	Surgery: removal of the abscess and contact with the transverse colon (about 10 cm), and the end-to-end anastomosis	N/A
Stefanidis K et al. (18)	2012	M	45	Middle transverse colon	I	lumbar pain	Duplication of the transverse colon	Plain X-ray: no abnormal finding; US: meaningless because the intestine is filled with gas; CT: a cystic structure (arrows) containing an air-fluid level; Barium enema: a large air-filled tubular structure in the left upper quadrant containing an air-fluid level	Surgery: cyst excision and anastomosis of colon colon	N/A
Ghosh JK et al. (19)	2015	M	48	Splenic flexure	III	Chronic diarrhea	Duplication of the transverse colon	Colonoscopy: two openings in the transverse colon; Barium enema: the lumen communicated with the transverse colon at both ends	Symptomatic anti-diarrhea treatment: rifaximin 550 mg twice daily for 14 days	6
Mourra N et al. (20)	2010	M	50	Middle transverse colon	I	Abdominal pain	Pancreatic cyst	CT and MRI showed pancreatic cyst; Ultrasound endoscopy: mesocolonic cyst	Surgery: complete excision of the cyst without colonic resection	N/A
Cavallaro G et al. (21)	2010	M	27	Transverse mesocolon	I	Acute and diffuse abdominal pain	Cystic lesion	CT and US: a cystic lesion of about 65 mm in diameter	Surgery: complete resection of the mass and a small section of the anterior colon wall	12
Shrestha S et al. (22)	2020	F	43	Distal transverse colon	I	Painful cystic swelling on the left upper and lumbar region extending to the central and right upper abdomen	duplication cyst of the colon	US: a 12 cm x 8 cm cystic lesion in the left lumbar region; Colonoscopy: normal findings; CECT: large cystic dilation of large bowel involving transverse colon with gas and faces indicating a possible diagnosis of mega-colon; Barium enema: a tubular duplication cyst of descending colon with a blind distal pouch attached to the retro-peritoneum.	Surgery: left hemicolectomy and excision of the duplication cyst	2
Li GB et al. (23)	2020	F	17	Splenic flexure	III	Constipation and chronic abdominal pain	Tubular colonic duplication	Barium enema: two enlarged loops with accumulated barium in the left lower quadrant; CT: two dilated lumen with a massive amount of stored feces in the left abdominal region	Surgery: a laparoscopic exploration and left hemi-colectomy	6
Peng Z et al. (24)	2023	F	52	Transverse mesocolon	I	Persistent upper abdominal pain, experiencing nausea, vomiting, and cessation of flatus and defecation	Colonic diverticulum	CT: a large, uniform, low-density shadow with a dilated intestinal tube and multiple air-fluid levels	Surgery: The cyst was decompressed, the transverse colon was disconnected 10 cm from the cyst, and a side-to-side anastomosis was performed.	6
Kim YW et al. (25)	2005	F	40	All of the transverse colon	III	No	Tubular duplication of the transverse colon	Barium enema: tubular duplication of the transverse colon; Colonoscopy: a normal colonic mucosa in the duplicated segment;	No	4
Zhang ZM et al. (26)	2022	M	18	Initiation of transverse colon	III	Recurrent intestinal obstruction	Megacolon	CT: distinct distention of the colonic lumen was identified; Colonoscopy: No structural abnormalities were found	Surgery: subtotal colectomy	10

F, Female; M, Male; CT, Computer Tomography; US, Ultrasound; MRI, Magnetic Resonance Imaging; CECT, Contrast-enhanced computed tomography.

There is a lack of unified diagnostic and treatment criteria for transverse colonic repetitive malformations. 12 cases were diagnosed using abdominal multirow CT (12, 13, 15–18, 20–24, 26), 8 cases using digitized barium enema colography (13, 14, 17–19, 22, 23, 25), and colonoscopy in 4 cases (19, 20, 25, 26), 8 cases using US (13–16, 18, 20–22) and 2 cases using plain X-ray (16, 18). Because the symptoms of repeat transverse colon malformations in adults are mostly nonspecific, clinical diagnosis is difficult. All diseases with abdominal pain and abdominal mass as the main complaints should be included in the differential diagnosis of this disease. The differential diagnosis of transverse colon duplication is shown in **Table 3**. Abdominal CT, barium enema, and colonoscopy remain the best diagnostic tools. US is often the first choice for screening because it has no radiation and is simple to operate. CT shows a comprehensive anatomical structure, and ^99^TC^M^-pertechnetate scintigraphy is helpful for the diagnosis of highly suspected intestinal duplication in clinical practice, especially for those with no clear positive performance on CT and ultrasound (30). Type I (simple cysts) are easier to observe on US than CT due to their liquid contents attached to the mesentery. Barium enema may show an open-mouth lesion that communicates with a normal bowel. Colonoscopy can also be used when repetitive malformations and the opening between the normal lumen are evident.

**Table 3:** Differential diagnosis of duplication of transverse colon.

Disease	clinical manifestation	Imaging examination	Treatment measure
Intestinal obstruction	Paroxysmal abdominal pain with nausea, vomiting, bloating and cessation of flatus and defecation	X-ray: presence of gas or liquid in the intestinal cavity; CT/MRI: The intestinal tube was inflated and dilated, and liquid gas flattening, intestinal wall thickening and intestinal diameter increasing were seen.	Fasting, gastrointestinal decompression, correction of water and electrolyte disorders and acid-base imbalance, prevention and treatment of infection and toxemia, ineffective conservative treatment can be considered surgical treatment.
Intussusception	Often occurs in children, paroxysmal abdominal pain, hematochezia, abdominal mass	X-ray: liquid level may be present; CT: characterized by concentric circles or target sign	In the early stage, it can be treated conservatively with enema therapy. In late stage or in cases where the enema has not been reduced, and in cases where the reduction has been more than 3 times, surgery is required.
Colon placeholder (benign and malignant tumors)	Changes in bowel habits, rectal bleeding or bloody stools, persistent abdominal discomfort	Barium enema: intestinal stenosis, mucosal fold disorder, destruction or disappearance, filling defects, etc Colonoscopy: The size, shape, color and location of the lesion can be observed, while biopsy is performed to make pathological diagnosis.	Endoscopic high-frequency electrocoagulation, laser and other methods can be used for resection, or surgical resection can be selected, and other treatments (such as radiotherapy, chemotherapy, surgery, etc.) can be selected for malignant transformation according to the situation.
Crohn's Disease	Diarrhea, abdominal pain, abdominal mass, etc	Barium enema: The lesions can involve the whole digestive tract, and the affected intestinal tubes show rough mucosa, intestinal rigidity, and segmental stenosis, showing a "linear" sign, and the lesions are distributed in a skipping manner. Endoscopy: discontinuous inflammatory changes were observed. Submucosal lymphoid follicles hyperplasia caused mucosal uplift and pebble-like changes.	Medication, lifestyle and diet changes, changes in eating habits, stress reduction, moderate exercise and exercise to prevent relapse and manage symptoms. Surgery is generally contraindicated and has not been shown to prevent remission.
Ulcerative colitis	Diarrhea, mucous, pus, blood stool, abdominal pain. Systemic symptoms may include weight loss, fever, anemia, hypoproteinemia and so on	Barium enema: multiple small ulcers; mucous membrane coarse or fine granular change; Colonoscopy: retrograde colitis, the colonic pocket may disappear, and the mucosal ulcer may be shallow	Control acute attack, drug maintenance remission, reduce recurrence, prevention and treatment of complications. Rest, diet and nutrition. Patients complicated with massive hemorrhage, intestinal perforation, severe megacolon, especially with toxic megacolon failed medical treatment, and patients complicated with cancer were treated with surgery.
Retroperitoneal tumor	There are no conscious symptoms in the early stage, and the tumor is often large when the symptoms are obvious. Clinical symptoms include abdominal distension, abdominal pain, and low back pain.	CT and MRI: the anatomical location, scope and size of the retroperitoneal space where the tumor is located can be determined. The pathological structure and type of the tumor can be determined.	Benign tumors should be surgically resected, and if they cannot be completely resected, intracapsular resection may be performed. Patients with malignant tumors without distant metastasis should strive for radical resection. When excision is not possible, chemotherapy or radiation therapy is selected based on biopsy results.
Omental cyst and mesenteric cyst	Abdominal mass, abdominal pain or feeling of falling, accompanied by diarrhea, wasting anemia, etc	US: Circular or semi-circular masses. The border is smooth, sharp, can also be petal-like halo; X-ray: soft tissue shadows can be seen; Barium enema: signs of intestinal compression and displacement; CT: Provides the best imaging diagnosis of cysts and can provide location determination	Cyst is small without treatment, cyst enlargement, easy to complicated with acute abdomen, once diagnosed, should be early surgery.
Abdominal abscess	Abdominal pain, fever and leukocytosis	CT: The initial abscess appears as a round or oval mass with lower density. Chronic abscess is characterized by low intermediate density and high peripheral density.	When the abscess is small or not formed, antibiotic therapy, systemic support therapy, physical dithermy, etc. When conservative treatment is ineffective or drainage is not smooth, surgical removal of pus and catheter drainage should be considered.

Surgical resection is a radical treatment for repetitive deformities of the colon. Only one patient in previous studies had been treated with drugs (19), with one case complicated by transverse colon carcinogenesis (12) and one case of mild dysplasia (20). Resection of repetitive malformations and additional resection of 2 cm to the normal bowel may disengage the fibrotic bowel and reduce the risk of perforation, bleeding, obstruction, and carcinogenesis (31). For type I, simple cystectomy and cyst mucosal decortication may be used, but if there is communication with the normal bowel tube, the adjacent bowel needs to be removed to ensure complete cyst resection. With the development of endoscopic technology, endoscopic submucosal dissection for the treatment of intestinal duplication has gradually become one of the new treatment methods that can partially replace surgical operations, which has the characteristics of less trauma and low cost. In recent years, ESD resection has also been reported (32).

## Conclusion

In conclusion, transverse colonic repetitive malformation is a rare gastrointestinal malformation with unclear pathogenesis. Children often have acute abdomen as the first symptom, while adults experience atypical onset and tumor compression symptoms such as abdominal mass, abdominal pain, and constipation. Full abdominal CT, contrast enema, and colonoscopy were usually used to make the diagnosis. Surgery is a radical treatment and reduces the possibility of perforation, bleeding, obstruction, and cancer.

## Data availability statement

The raw data supporting the conclusions of this article will be made available by the authors, without undue reservation.

## Ethics statement

Written informed consent was obtained from the [individual(s) AND/OR minor(s)’ legal guardian/next of kin] for the publication of any potentially identifiable images or data included in this article.

## Author contributions

XZ and GD conceptualized the report. GG supervised the data collection. XZ and GD drafted the report. All authors contributed to the article and approved the submitted version.
